# Discrete Geodesic Distribution-Based Graph Kernel for 3D Point Clouds

**DOI:** 10.3390/s23052398

**Published:** 2023-02-21

**Authors:** Mehmet Ali Balcı, Ömer Akgüller, Larissa M. Batrancea, Lucian Gaban

**Affiliations:** 1 Department of Mathematics, Faculty of Science, Muğla Sıtkı Koçman University, 48000 Muğla, Turkey; 2 Department of Business, Babeş-Bolyai University, 7 Horea Street, 400174 Cluj-Napoca, Romania; 3 Faculty of Economics, “1 Decembrie 1918” University of Alba Iulia, 510009 Alba Iulia, Romania

**Keywords:** simplicial complex, Wasserstein distance, Kullback–Leibler information, point cloud processing

## Abstract

In the structural analysis of discrete geometric data, graph kernels have a great track record of performance. Using graph kernel functions provides two significant advantages. First, a graph kernel is capable of preserving the graph’s topological structures by describing graph properties in a high-dimensional space. Second, graph kernels allow the application of machine learning methods to vector data that are rapidly evolving into graphs. In this paper, the unique kernel function for similarity determination procedures of point cloud data structures, which are crucial for several applications, is formulated. This function is determined by the proximity of the geodesic route distributions in graphs reflecting the discrete geometry underlying the point cloud. This research demonstrates the efficiency of this unique kernel for similarity measures and the categorization of point clouds.

## 1. Introduction

Point clouds are one of the most direct representations of geometric datasets. One of the sources for obtaining point clouds is 3D shape acquisition devices such as laser range scanners, which also have applications in many disciplines. These scanners provide generally noisy raw data in the form of disorganized point clouds representing surface samples. Given the growing popularity and very wide applications of this data source, it is essential to work directly with this representation without having to go through an intermediate step that can add computational complexity and fitting errors. Another important area where point clouds are frequently used is the representation of high-dimensional manifolds. Such high-dimensional and general isodimensional data are found in nearly all disciplines, from computational biology to image analysis and financial data [1,2,3,4,5]. In this case, due to the high dimensionality, manifold reconstruction is challenging from a technological standpoint, and the relevant calculations have to be performed directly on the raw data, i.e., the point cloud.

Graph structures are important tools for representing discrete geometric data and the discrete manifold underlying the data, as they can reflect the structural and relational arrangements of objects [6,7,8,9]. In the classification of discrete graph-based geometric structures, the problem of accurately and effectively calculating the similarity of these data sets arises. Graph kernel functions are widely used to solve this type of problem [10,11,12,13,14]. Graph kernels have proven to be powerful tools for the structural analysis of discrete geometric data. There are two main advantages to using graph kernel functions. First, graph kernels can characterize graph properties in a high-dimensional space and therefore have the capacity to preserve the topological structures of the graph. Second, graph kernels make rapidly evolving machine-learning methods for vector data applicable to graphs.

The goal of this study was to construct a kernel function that generalizes across the topology and geometry of a 3D point cloud. A geodesic is a curve in differential geometry that depicts the shortest route between two points on a surface, or more broadly, the shortest path on a Riemannian manifold. In addition, it is intended to expand the idea of a ”straight line” on any differentiable manifold coupled to a more generic medium. Geodesics on piecewise linear manifolds, the foundation of discrete differential geometry, were first defined by [15] and then extended to polyhedral surfaces by [16]. In discrete differential geometric techniques, the underlying geometry of the point cloud is assumed to be known, and triangulation is used to include the locations between points into the geometry. Thus, noise reduction and subdivision techniques may also be implemented while using a raw 3D point collection. In the graph structure to be constructed from the 3D point cloud, the idea of a geodesic becomes the problem of the shortest path [17,18].

In general, point clouds should intensively sample the border of a smooth surface rebuilt using moving least squares [19,20,21], implicit [22], or Voronoi/Delaunay [23,24] methods. Since point clouds may describe 3D forms using graphs without the requirement for the explicit storing of the manifold connection, they have become a popular alternative surface representation to polygonal meshes [25,26,27,28]. Despite the fact that numerous particular graph types, such as the *k*-nearest neighbor graph [29], the Reeb graph [30], and the Gabriel graph [31], give methods to the geometry and topology of point clouds, higher-order topologies and submanifold topologies disregard topological characteristics. Graph representations of simplicial complex skeletons that preserve the submanifold topologies of point clouds and may potentially integrate higher-order topological information are used in this study. In general, skeleton-based representations provide a compact and expressive form abstraction that aims to imitate human intuition. Using concise, informative, and easily computable skeletal representations as opposed to full models may facilitate the comparison process. In practice, it may be hard to locate a query-like item in a database by comparing point clouds or stacks of hundreds of triangles.

This work presents an approach to the geodesic curves of the manifold by calculating the shortest paths on the graph structures defined by the simplicial complex skeleton of the submanifold from which the point cloud is taken. If the graph structures created by the simplicial complex skeleton are altered by noise, the approaches to the geodesic distributions of the submanifold are not significantly impacted. Consequently, a kernel function to be defined by the Wasserstein similarity of the distributions of discrete geodesics in the skeletons of 3D point clouds has shown to be an excellent assessment tool for point cloud similarity. In a variety of 3D applications, such as 3D object retrieval and inverse procedural modeling, measuring the similarity between 3D geometric objects is crucial. This study aimed to find and demonstrate the effectiveness of a kernel function that calculates the similarity of 3D point clouds while taking into consideration the discrete geometry and topology of the point cloud. The effective kernel function is obtained using the Wasserstein-1 distance by comparing the geodesic distributions in the graphs to those that are isomorphic to the skeleton of the simplicial complexes on the point cloud.

Direct comparison and classification are utilized to compare this newly constructed kernel function to graph models. Using the values of the kernel function, the intra-class and inter-class similarity matrices of the point clouds were constructed during the direct comparison procedure. Considering the geometric and topological aspects of the studied models, it has been noted that the Alpha complex skeletons are the networks on which the kernel function operates most effectively. Using the kernel function, the similarity of a point cloud to itself was stored in the matrices used in the classification procedures. At this, the graph communities acquired for each point are used. Using a convolutional neural network, supervised classification is performed according to the airplane, car, person, plant, and vase classes.

### Related Works

As a collection of large points in three dimensions, point clouds may stand in for an object’s spatial distribution and surface properties. Model reconstruction [32,33], terrain monitoring [34,35], and resource monitoring and exploitation [36,37] are just a few examples of the various research and application sectors that rely on 3D point clouds obtained by stationary laser scanning. Accurate and efficient registration is crucial for obtaining a full scene or object from a collection of point clouds obtained via stationary laser scanning [34,38], and this has a knock-on effect on subsequent processing and applications such as segmenting and classifying the cloud as well as detecting and tracking objects within it. Moreover, semantic segmentation using point cloud data has made significant strides in recent years [39,40,41,42,43], thanks in large part to innovations such as point-cloud compression techniques [44,45]. When separating moving objects from LiDAR point clouds, semantic segmentation is essential. Existing semantic segmentation convolutional neural networks are good at predicting the semantic labels of point clouds such as automobiles, buildings, and people. Hence, new computational techniques on point clouds are needed because of their widespread use in many disciplines.

The kernel function of a pair of graphs may be described by decomposing the graphs and comparing the specified pairings of isomorphic substructures. Commonly used substructures are walks, paths, and spanning trees [46,47,48]. In [49], the authors evaluated the complex motions as decomposed spatio-temporal parts for each video using kernel functions defined with subtrees and created the corresponding binary trees. The resulting kernel function is defined by calculating the number of isomorphic subtree patterns. A kernel family to compare point clouds is proposed by [50]. These kernels are based on a newly developed local tree-walk kernel between subtrees, which is defined by factoring in the properly defined graph models of subtrees. The authors in [51] defined a graph kernel for motion recognition in videos. First, they describe actions in videos using directed acyclic graphs (DAGs). The resulting kernel is defined as an expanding random walking kernel by counting the number of isomorphic walks of the DAGs. [52] proposed a segmentation graph kernel for image classification. In this method, each image is represented by a segmentation graph; each vertex corresponds to a segmented region, and each edge connects a pair of neighboring regions. The resulting kernel function is calculated by counting the imprecise isomorphic subtree patterns between the segmentation graphs. Furthermore, some kernel functions are also effectively used for computer vision applications, such as the shortest path graph kernel [53], non-backward walking kernel [54], Lovas kernel [55], Weisfeiler–Lehman subtree kernel [56].

Although each of the kernel functions mentioned above gives effective results for different problems, they are very sensitive to noise and outliers in empirically obtained 3D point clouds. Furthermore, they do not generate reliable comparison information between isomorphic substructures. In other words, for graphs abstracted from 3D shapes, most of the available kernels cannot determine whether isomorphic substructures are located in the same regions based on the visual background. To overcome these shortcomings, [57] proposed an aligned subtree kernel. The proposed kernel function is calculated by counting the number of isomorphic subtrees rooted in aligned vertices, thus overcoming the shortcoming of neglecting positional or structural correspondences between isomorphic substructures that arise in most graph kernels. Although an aligned subtree kernel is effective on 3D shape classification problems, it cannot guarantee transitivity between aligned vertices. More specifically, given the vertices *u*, *v*, and *w*, if *v* and *u* and *u* and *w* align, the kernel function cannot guarantee that *v* and *w* are also aligned. On the other hand, [58] shows that the cascading alignment step is necessary to guarantee the positive precision of the vertex alignment kernel. Therefore, the aligned subtree kernel cannot be guaranteed as a positive-definite kernel. Furthermore, all the specified kernels reflect only graph properties for each graph pair under comparison and therefore ignore information from other graphs. These disadvantages limit the precision of kernel-based similarity measures.

This paper is organized as follows: In Section 2, we first present the basics of simplicial complexes and graph data obtained from their 1-skeleton. The method of obtaining the graph defined in this way is very important for the kernel function presented, since it will most effectively use the topological and geometric properties of point clouds. Then, the distributions of discrete geodesic curves on these graph structures are determined using Kullback–Leibler information and then a kernel function using the Wassertein-1 distance is introduced. In Section 3, we give basic computational results for this novel kernel function on the Princeton ModelNet-40 benchmark. The computational results measure the similarity of point clouds with each other and show the classification of point clouds. Finally, in Section 4, we present detailed conclusions.

## 2. Methodology

### 2.1. Simplicial Complexes

The convex body of the points with $v_{0},v_{1},\ldots ,v_{d}\in R^{n}$ being d+1-affine independent points is called a *d*-symplex. The details about simplicial topology can be found in [59]. In this study, a *d*-simplex is denoted by $\sigma =conv\left\{v_{0},\ldots ,v_{n}\right\}=\left[v_{0},\ldots ,v_{n}\right]$. The convex body is simply a polyhedron with d+1-affine independent points as vertices. The face of a σ is defined by conv{S} as $S\subset [v_{0},\ldots ,v_{n}]$. The finite family of simplexes that provide the following properties is called a *K* complex:

- For σ∈K and τ is face of σ, τ∈K;
- When $\sigma ,\sigma^{\prime }\in K$, $\sigma \cap \sigma^{\prime }$ is empty or is simultaneously a face of σ and $\sigma^{\prime }$.

**Figure 1:**
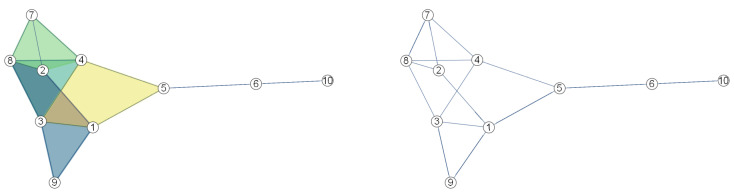
A 2-dimensional complex (**left**) and its 1-skeleton (**right**), where the numbers denote the vertex index.

A collection {σ∈K | dim(σ)≤j} of a simplicial complex *K* is called the *j*-skeleton of *K* and is denoted by $K^{\left(j\right)}$. In this definition, dim(σ) denotes the dimension of σ. The 1-skeleton of any *K* complex is $K^{\left(1\right)}=\left\{\sigma \in K\;|\;dim\left(\sigma \right)\leq 1\right\}$, that is, the set of vertices and edges of the simplex that form the complex. Hence, for $V=\{v_{0},\ldots ,v_{n}\}$ and $E=\{\left[v_{i},v_{k}\right]\;|\;\left[v_{i},v_{k}\right]\in K\}\subseteq V\times V$, there exists a simple graph G=(V,E) with $K^{\left(1\right)}\equiv G$. An example of a 2-dimensional *K* complex and the corresponding $K^{\left(1\right)}$ skeleton is given in Figure 1. It is straightforward to see that $K^{\left(1\right)}\equiv G=\left(V,E\right)$ with $V=\{v_{1},\ldots ,v_{10}\}$.

With a *K* complex to be formed on a 3D point cloud, it is possible to capture the topological features of the underlying manifold of the point cloud, such as connectivity and holes. Moreover, it is possible to apply various graph algorithms to the simple graph which the skeleton $K^{\left(1\right)}$ is isomorphic. Let us consider the shortest graph paths $\ell_{1}=v_{7},v_{8},v_{3},v_{9}$ and $\ell_{2}=v_{7},v_{2},v_{1},v_{9}$ between the vertices $v_{7}$ and $v_{9}$ on the $K^{\left(1\right)}$ skeleton in Figure 1. It is obvious that the $\ell_{1}$ and $\ell_{2}$ are homotopic on $K^{\left(1\right)}\equiv G$. Thus, when comparing two skeletons $K_{1}^{\left(1\right)}\equiv G_{1}$ and $K_{2}^{\left(1\right)}\equiv G_{2}$ in terms of topological similarity, examining the approximation distributions of discrete geodesics corresponding to the shortest paths yields effective results.

Let us now consider the methods of obtaining various geometric or abstract complexes, which are widely used in practice. In particular, the input is often a series of points from which some hidden field is sampled or approximated. This set of points is called a point cloud. Point clouds have no topology other than a discrete topology, and some connections and some topologies are applied to them. Let a point cloud be $P=\{p_{1},\ldots ,p_{n}\}$ and the Euclidean ball be B(p,r) with $p_{i}\in P$ at the center and *r* is the radius. We introduce the simplicial complex forming algorithms that we discussed in this study as follows:

**Definition** **1.** *For a point cloud $P\subset R^{3}$, a simplex $\sigma =[p_{i_{0}},\ldots ,p_{i_{d}}]$ is in the Delaunay complex Del(P) if and only if there is a ball B whose boundary contains the vertices of σ and does not include the other points of the point cloud.*

**Figure 2:**
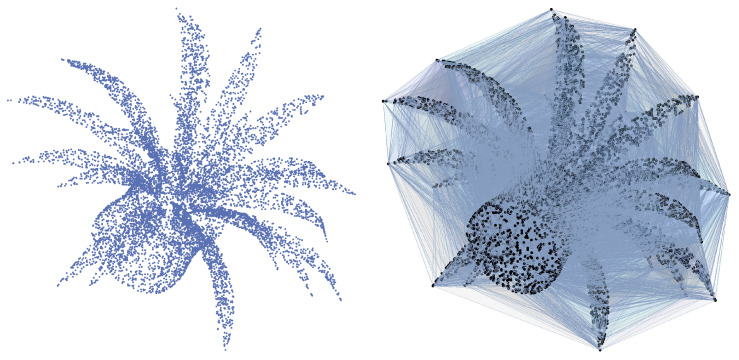
A point cloud example (**left**) and its Delaunay complex (**right**).

As an indicative for the simplicial complexes employed in the research, Figure 2 provides a point cloud and an example of the Delaunay complex derived from it.

Delaunay complexes have very rich geometric features in 2D and 3D [60,61]. Only computing first-dimensional Delaunay complexes does not seem to be asymptotically faster than the computation of the full Delaunay complex. This makes the complex less attractive for high-dimensional data analysis. In this situation, Delaunay complexes’ subcomplex Alpha complexes become a key tool for topological data processing. The Delaunay triangulation of a point cloud includes a subset of the faces, which together make up the Alpha complex $A_{r}\left(P\right)$, a *d*-dimensional simplicial complex [62].

**Definition** **2.** *For a point cloud $P\subset R^{3}$ and the given real number r>0, a simplex $\sigma =[p_{i_{0}},\ldots ,p_{i_{d}}]$ is in the Alpha complex $A_{r}\left(P\right)$ if and only if $\underset{p\in Q\subset P}{⋂}Vor_{r}\left(p,P\right)\neq \emptyset$, where $Vor_{r}\left(p,P\right)$ is the Voronoi ball of p defined by the intersection of the open ball centered at p with radius r and the Voronoi cell of p∈P.*

**Definition** **3.** *For a point cloud $P\subset R^{3}$ and the given real number r>0, a simplex $\sigma =[p_{i_{0}},\ldots ,p_{i_{d}}]$ is in the Čech complex $C_{r}\left(P\right)$ if and only if $\underset{0\leq j\leq d}{⋂}B\left(p_{i_{j}},r\right)\neq \emptyset$.*

It should be noted that the definition of the Čech complex includes a parameter *r*, which may be useful in practice. Specifically, we can think of creating a sphere at each $p_{i}$ in the point cloud and looking at the convergence of the spheres on the *r* scale.

**Definition** **4.** *For a point cloud $P\subset R^{3}$ and the given real number r>0, a simplex $\sigma =[p_{i_{0}},\ldots ,p_{i_{d}}]$ is in the Vietoris Rips complex $VR_{r}\left(P\right)$ if and only if $\forall j,j^{\prime }\in \left[0,d\right]$, $B\left(p_{i_{j}},r\right)\cap B\left(p_{i_{j}^{\prime }},r\right)\neq \emptyset$.*

In other words, the points $p_{i_{0}},\ldots ,p_{i_{k}}$ span a *d*-simplex if and only if the Euclidean balls with radius *r* centered at these points have a pairwise intersection.

**Definition** **5.** *Let ∀i∈[0,d] and $q\in Q=P\setminus \{q_{0},\ldots ,q_{d}\}$. If $d\left(q_{i},x\right)\leq d\left(q,x\right)$, then it is said that the simplex $\sigma =[q_{0},\ldots ,q_{d}]$ is weakly witnessed by point x and if $d\left(q_{i},x\right)=d\left(q,x\right)$, then it is said that the simplex $\sigma =[q_{0},\ldots ,q_{d}]$ is strongly witnessed by the point x. For a point cloud $P\subset R^{3}$ and Q⊂P, the witness complex W(Q,P) is the simplicial complex whose vertices are from the set Q and all faces are weakly witnessed by a point in P.*

Detailed information for these complexes, which are frequently used in the literature, can be found in [63,64,65]. Moreover, the following features can be given from the same studies:

(1) W(Q,P)⊆Del(P)

and

(2) $$C_{r}\left(P\right)\subseteq VR_{r}\left(P\right)\subseteq C_{2c}\left(P\right).$$

A comparison for the complexes used in this study is given in Table 1.

In this study, temporal modified methods introduced by [66,67] are used for Vietoris Rips and Witness complexes, respectively. The time complexities of these methods are given in Table 1. M(|P|) represents the complexity of the product of a matrix of type |P|×|P| and μ represents the time complexity of the sparsity function of the marker of the subset *Q*.

### 2.2. Kernel Function

Modeling and computing object similarity is one of the most difficult tasks in machine learning. When it comes to graphs, graph kernels have gotten a lot of press in recent years and have emerged as the most popular method for learning from graph-structured data. A graph kernel is a symmetric, positive semi-definite function defined on the space of graphs. This function can be expressed as an inner product in some Hilbert spaces. In particular, given a kernel κ, there exists a transformation φ:G→H mapping a graph space G to a Hilbert space H such that for each $G_{1},G_{2}\in G$, $\kappa \left(G_{1},G_{2}\right)=<\varphi \left(G_{1}\right),\varphi \left(G_{2}\right)>$.

Graph kernels handle the challenge of graph comparison by attempting to efficiently capture as much of the graph’s topology as possible. One of the primary reasons for the widespread adoption of graph kernel methods is that they enable a vast array of techniques to operate directly on graphs. Thus, graph kernels enable the application of machine learning methods to real-world situations using graph-structured data.

First, information on the distributions will be presented before the definition of a kernel function κ defined by the distribution of the geodesics of the graphs $K_{1}^{\left(1\right)}\equiv G_{1}$ and $K_{2}^{\left(1\right)}\equiv G_{2}$.

Kullback–Leibler information is a measure of how far apart two pieces of information are in terms of probability [68]. Let P and Q be two probability measures with densities of *p* and *q*, respectively. Then, the Kullback–Leibler information is defined by

(3) $$L_{X}\left(P,Q\right)=\int \log\left(\frac{p(x)}{q(x)}\right)p\left(x\right)d\mu \left(x\right)=E_{p}\left[\log\left(\frac{p(x)}{q(x)}\right)\right].$$

Since Kullback–Leibler information is not symmetric, it is not a metric. However $L_{X}\left(P,Q\right)+L_{X}\left(Q,P\right)$ is symmetric and is called the Kullback–Leibler divergence [69].

Let us consider a random variable *X* with a mean $\mu_{X}$ and a metric function defined by $d\left(x;\mu_{X}\right)=f\left(x-\mu_{X}\right)$. For $\mu_{X},\mu_{X}^{\prime }\in R$, a function defined by $g\left(x;\mu_{X}\right)=d\left(x;\mu_{X}^{\prime }\right)-d\left(x;\mu_{X}\right)$ is Jensen equal; that is, $E\left[g\left(x\right)\right]=g\left(E[x]\right)$. Let c∈R and $\left(\nu ,\omega \right)\in R\times R^{-}$. For a function $f(x;\mu_{X})$ with E[X]=c, with the density function

(4) $$f\left(x;\mu_{X}\right)=\nu \exp\left(\omega d\left(x;\mu_{X}\right)\right)=g\left(d\left(x;\mu_{X}\right)\right)$$

$L_{X}(\left(P,Q\right)$ becomes a metric [70].

Let us look at how to use Kullback–Leibler information to explain geodesic distributions on graphs. The geodesic distance between two vertices *u* and *v* in a graph *G* is defined as the number of edges of the shortest path linking *u* and *v* and denoted as $d_{G}\left(u,v\right)$. For example, in Figure 1, $d_{K^{\left(1\right)}}\left(2,5\right)=2$. The greatest geodesic distance between *u* and any other vertex is called the eccentricity of *u* and denoted by ε. It can be thought of as a measure of how far a vertex is from the furthest vertex in the graph. The eccentricity property allows the radius and diameter of a graph to be defined. The radius of a graph is the minimum eccentricity between the vertices of the graph. The diameter of a graph is the maximum eccentricity of any vertex of the graph. Hence, the diameter is the greatest distance between any pair of vertices. In order to find the diameter of a graph, we first find the shortest path between each pair of vertices using the landmark-based method presented in [71]. The greatest distance of any path is the diameter of the graph. Using the eccentricity of a graph, it is possible to define its two subgraphs. A central subgraph of $K^{\left(1\right)}$ is the graph with *n* vertices of degree α and the smallest eccentricity, and is denoted by $K_{n,\alpha }^{(1),C}$. An orbital subgraph of $K^{\left(1\right)}$ is the graph with *n* vertices of degree β and with *n* vertices and is denoted by $K_{n,\beta }^{(1),O}$.

Considering the Kullback–Leibler divergence, we can define two types of geodetic distributions for Jensen equal functions:

**Definition** **6.** *Central geodesic density function of $K^{\left(1\right)}$ is*

(5) $$f_{C}\left(u,\alpha ,\nu \right)=\nu \exp\left(-d_{G}\left(u,K_{n,\alpha }^{(1),C}\right)\right),$$

*and the orbital geodesic density function of $K^{\left(1\right)}$ is*

(6) $$f_{O}\left(u,\beta ,\nu \right)=\nu \exp\left(-d_{G}\left(u,K_{n,\beta }^{(1),O}\right)\right),$$

*where ν≥1 is a normalization factor.*

On a space of probability measures, the Wasserstein distances give a natural metric. They intuitively assess the least amount of effort necessary to change one distribution into another. The Wasserstein distances, in general, do not permit closed-form formulations; however, for R, we have the explicit form as

(7) $$W_{1}\left(\rho_{1},\rho_{2}\right)=\int_{R}\left|F_{\rho_{1}}\left(t\right)-F_{\rho_{2}}\left(t\right)\right|\;dt,$$

where $F_{\rho_{1}}\left(t\right)$ and $F_{\rho_{2}}\left(t\right)$ are the cumulative distribution functions of $\rho_{1}$ and $\rho_{2}$ [72]. It is feasible to compare the geodesic distributions with the kernel function below using the Wasserstein-1 distance function:

**Definition** **7.** *Let $K_{1}^{\left(1\right)}=\left(V_{1},E_{1}\right)$ and $K_{2}^{\left(1\right)}=\left(V_{2},E_{2}\right)$. Then, we have a kernel function*

(8) $$\kappa_{J}\left(K_{1}^{\left(1\right)},K_{2}^{\left(1\right)}\right)=\frac{W_{1}\left(\cup_{i=1}^{|V_{1}|},f_{C}\left(u_{i},\alpha_{1},\nu_{1}\right),\cup_{j=1}^{|V_{2}|},f_{C}\left(u_{j},\alpha_{2},\nu_{2}\right)\right)}{W_{1}\left(\cup_{i=1}^{|V_{1}|},f_{O}\left(u_{i},\beta_{1},\nu_{1}\right),\cup_{j=1}^{|V_{2}|},f_{O}\left(u_{j},\beta_{2},\nu_{2}\right)\right)},$$

*where $W_{1}$ is Wassterstein-1 distance.*

We shall note that the kernel function defined in Equation (8) is symmetric and positive definite.

## 3. Results

### 3.1. Data Set

Princeton ModelNet (Internet Access: https://modelnet.cs.princeton.edu/, accessed on 10 January 2023) seeks to give researchers in computer vision, computer graphics, robotics, and cognitive science a thorough and organized library of 3D CAD models. The researchers used information from the SUN database to create a list of the most widespread object types on the globe, which served as the foundation of the dataset. After developing an object vocabulary, the 3D CAD models of each item category were gathered by searching web search engines for each category phrase. Then, using custom-made tools with quality control, human employees were hired at Amazon Mechanical Turk to manually determine whether each CAD model fits into the designated categories [73].

Two methods are used to assess the effectiveness of the graph kernel function given in this paper. The first strategy is to assess the extent to which the topologies of the underlying point cloud geometries have an impact on the kernel function. In this study, the kernel function is used to determine the extent to which point clouds are similar or dissimilar. Point cloud samples containing 10,000 points are taken from the ModelNet-40 dataset for the first method. These point cloud examples belong to the airplane, car, person, plant, and vase classes as they have different inter- and intra-class topologies. Ten sample point clouds are taken from each class, and the kernel function presented in this study is run between these samples.

The second strategy involves using the provided kernel function to create a classification that is based on machine learning. On the ModelNet-40 dataset, this classification methodology is used, and it is compared to other approaches.

### 3.2. Point Cloud Comparison

**Figure A1:**
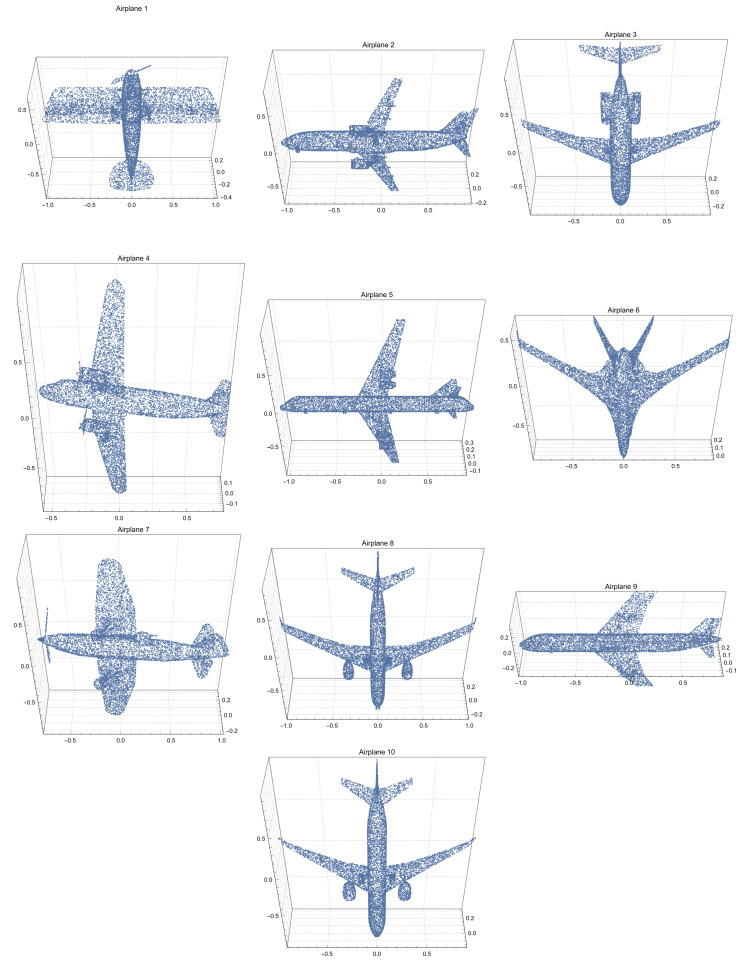
Airplane Models.

**Figure A2:**
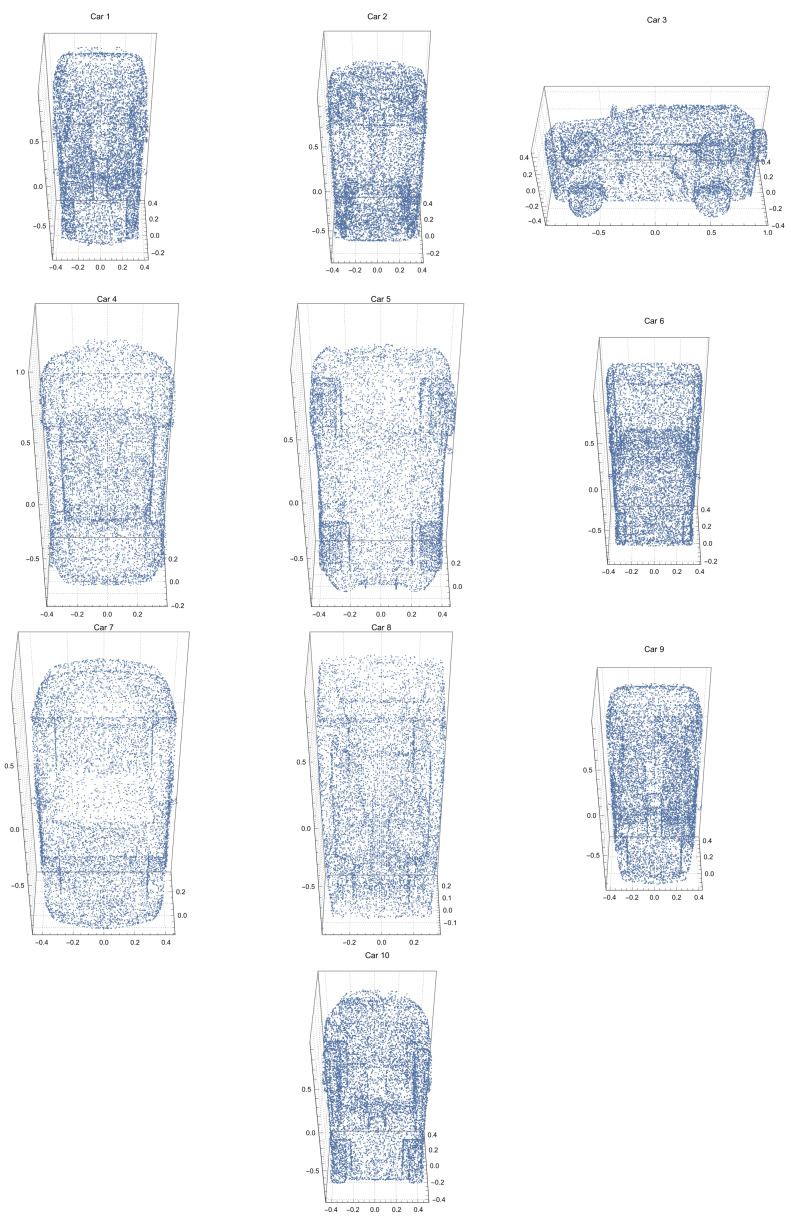
Car Models.

**Figure A3:**
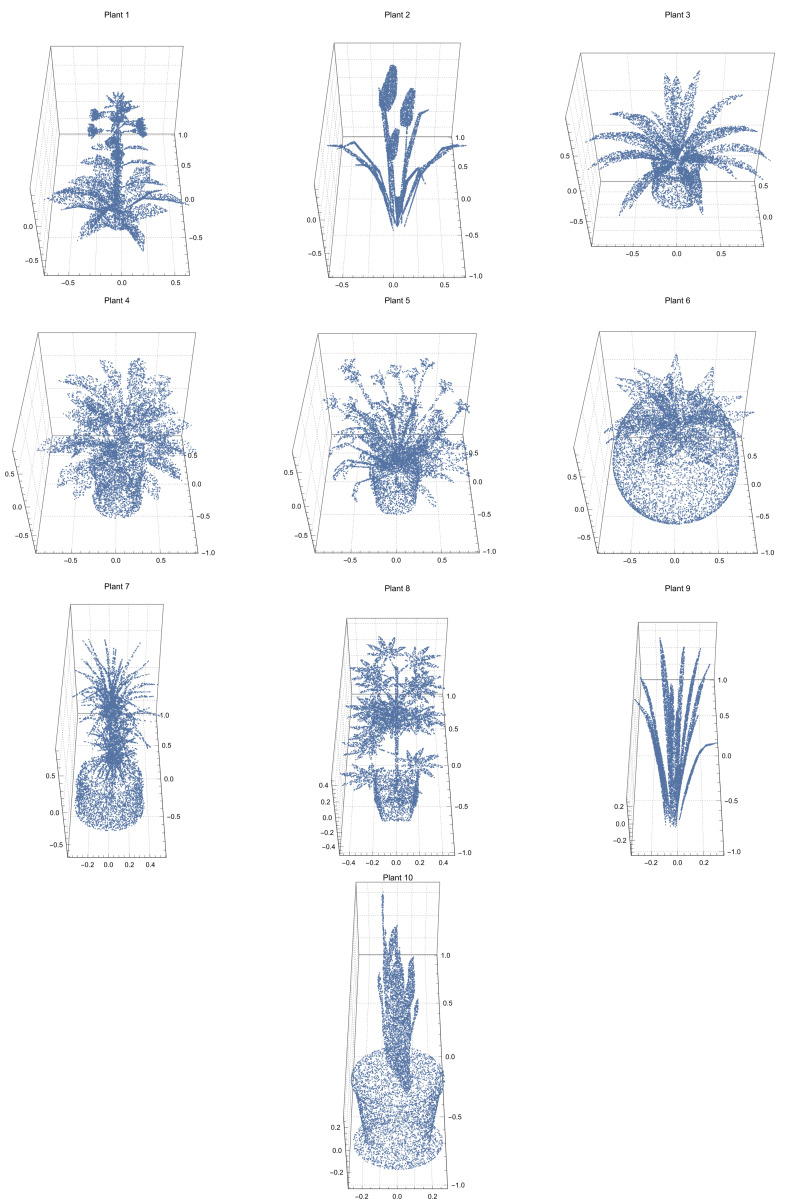
Plant Models.

**Figure A4:**
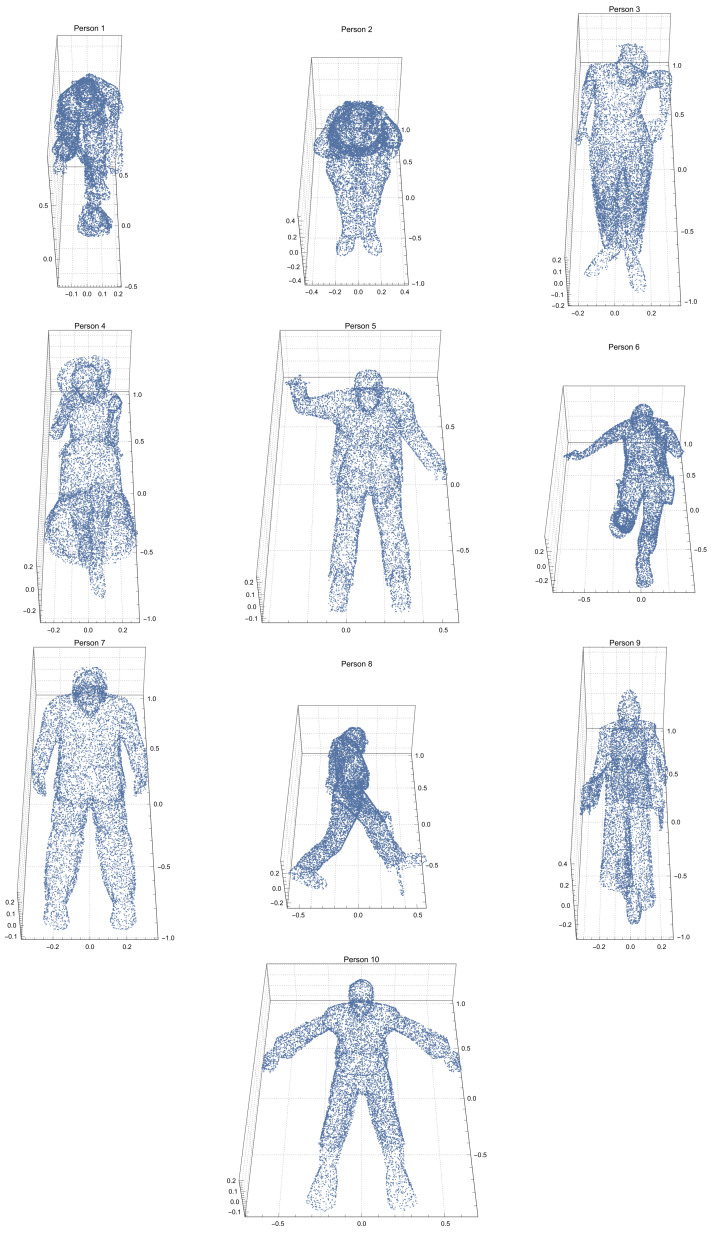
Person Models.

**Figure A5:**
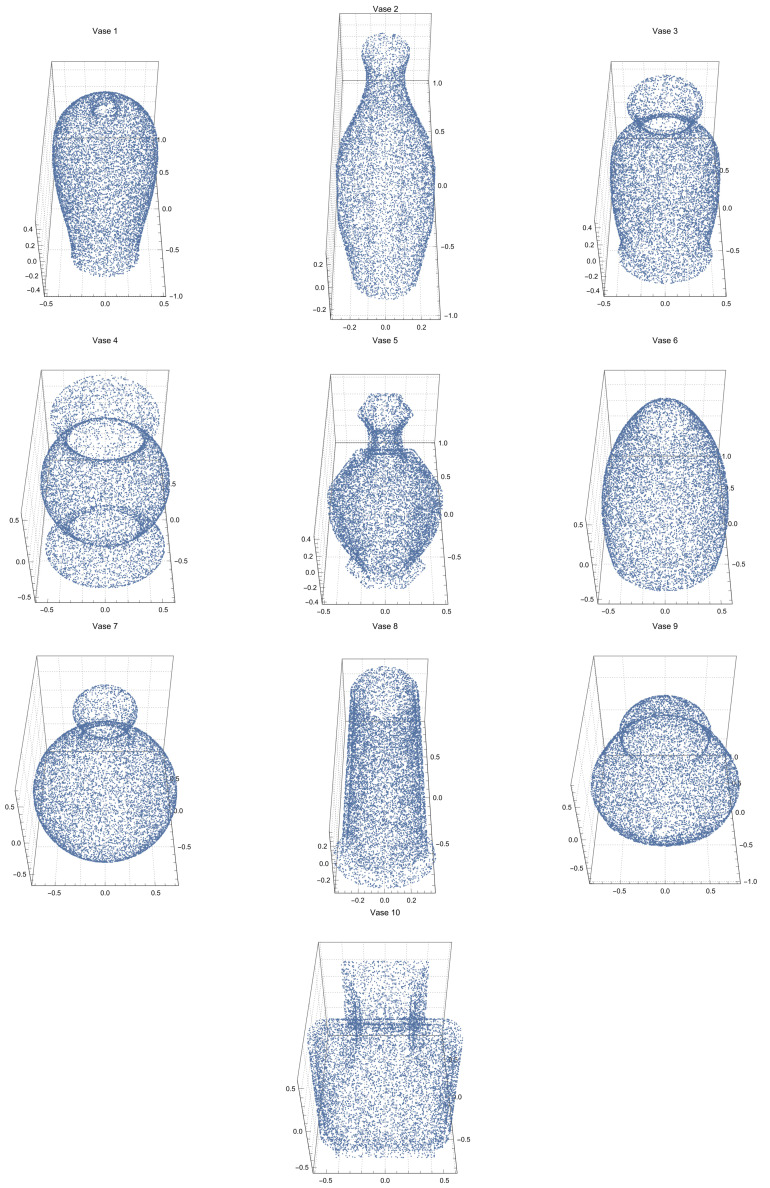
Vase Models.

To produce graphs from point clouds, each of the simplicial complex derivation techniques described in Section 2.1 are applied to the sample set, and the 1-skeletons of the complexes were selected as the representation graph. Equation (8) was used in this part to generate the graphs for each data group as well as the kernel matrices of the data subgroups. The point clouds used in this approach are presented in Figure A1, Figure A2, Figure A3, Figure A4 and Figure A5.

The relevant parameters are selected in the formation of simplicial complexes as the minimum values that connect the 1-skeletons derived from the point cloud. Using a step size of h=0.001 and beginning at 0, the least parameter is found. It is anticipated that different parameters will be found for each sample, both inter- and intra-classes. In addition to this, no discernible variation in the computational time complexity of the simplicial complexes’ generation procedures was found. Moreover, better computational times could result from differentiating the approach taken when determining the lowest parameter.

**Figure 3:**
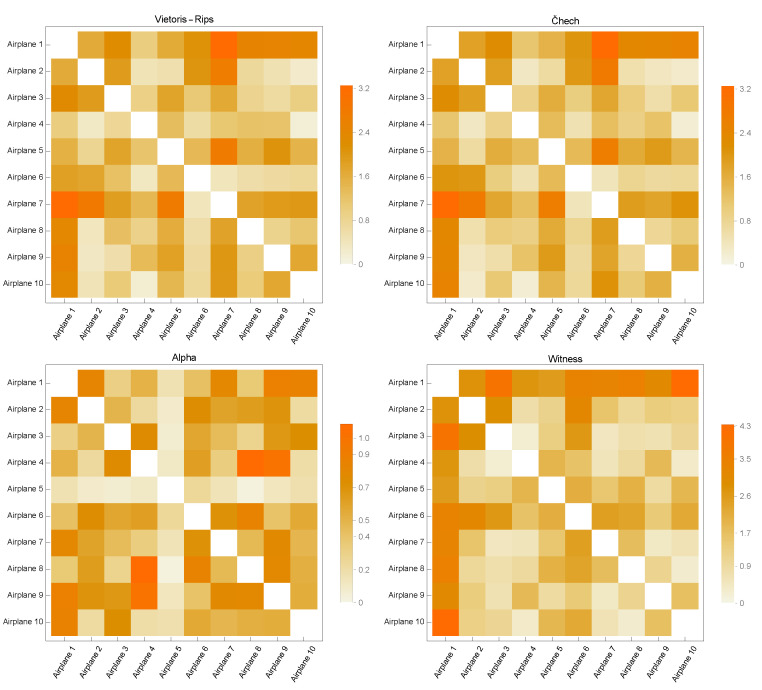
Similarity Matrices for Airplane Models.

**Figure 4:**
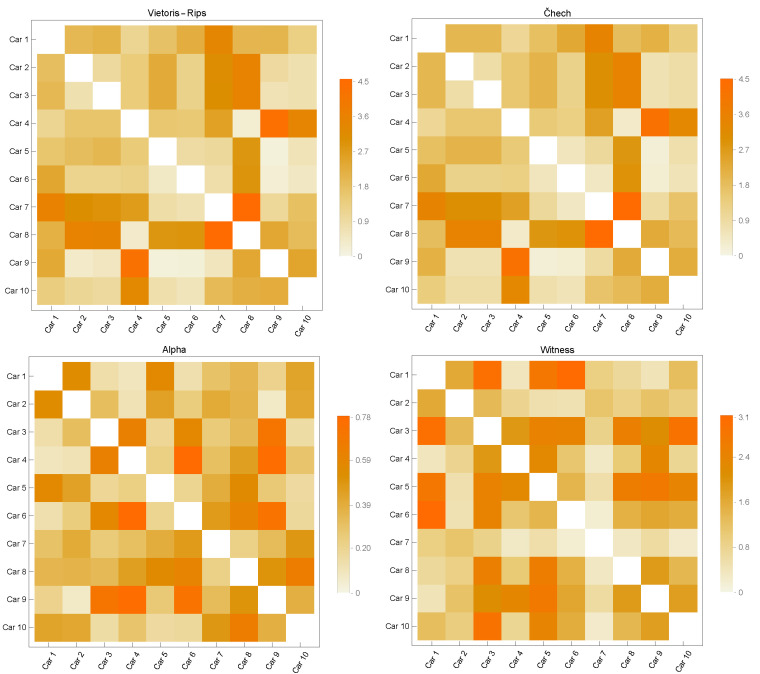
Similarity Matrices for Car Models.

**Figure 5:**
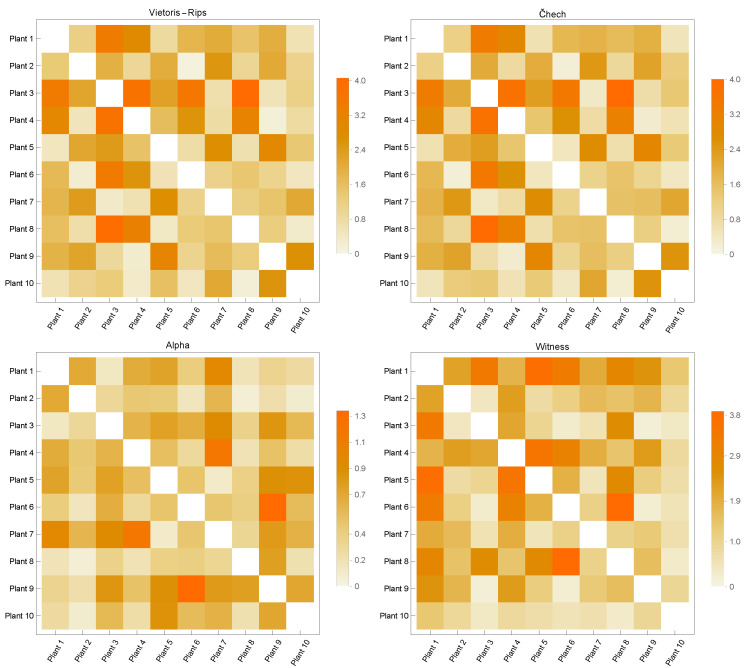
Similarity Matrices for Plant Models.

**Figure 6:**
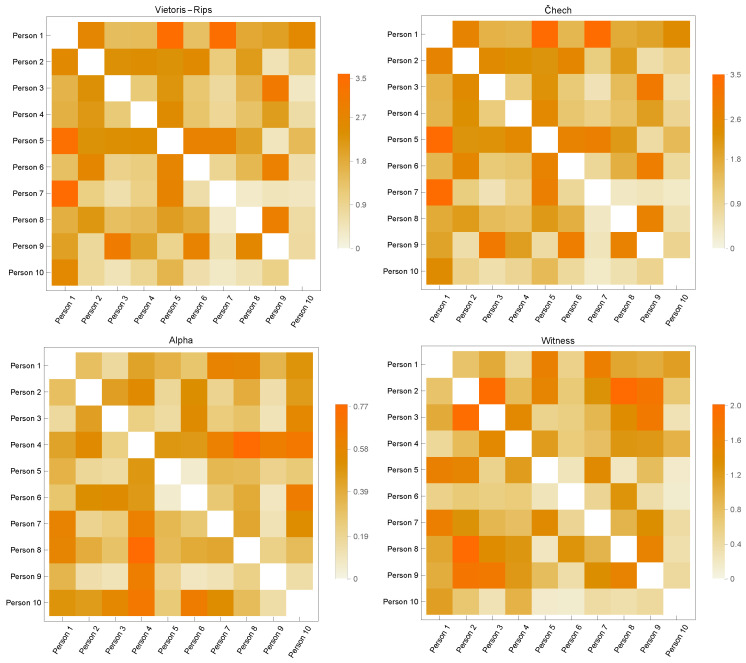
Similarity Matrices for Person Models.

**Figure 7:**
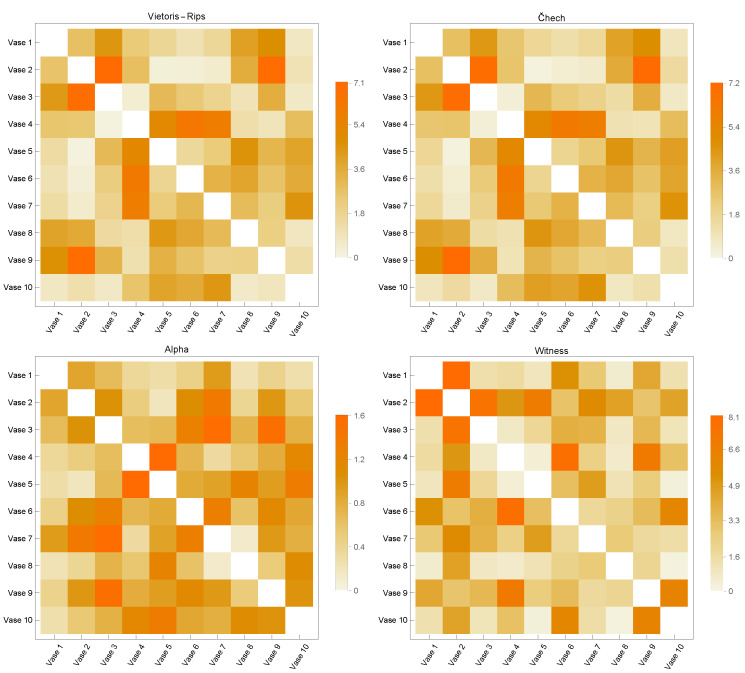
Similarity Matrices for Vase Models.

Figure 3, Figure 4, Figure 5, Figure 6 and Figure 7 contain similarity matrices obtained by using the kernel function to compare point clouds with each other. The samples from each class are complexed using the previously discussed simplex techniques, and the kernel function is applied to the graph structures that were built using the 1-skeletons of the samples.

Similarity matrices generated using the kernel function encode the measurements of the similarity between the point clouds. In the context of the distribution of discrete geodesics, the near-zero values of the kernel function provided by Equation (8), whose inputs are two graphs produced from point clouds, indicate that the two graphs are highly similar. Likewise, the graphs diverge in terms of similarity within the same context for large numbers. This study used four of the most fundamental simplicial complex approaches to determine the similarity metrics within each class. The analysis of the similarity matrices reveals that the common points of the point clouds in the discrete geometry environment within each class are most evident in the graphs produced with Alpha complexes. The geometries underlying the point clouds of the Airplane models are most comparable in the case of the Airplane 5 model. The matrix given in Figure 3 clearly demonstrates that the similarity value derived from the graph of Alpha complexes for this model is close to zero. When a comparable course is followed, the Airplane 4 and 8 models entirely diverge in terms of similarity, as shown by the matrix’s high values. In the similarity matrices provided in Figure 4, Figure 5, Figure 6 and Figure 7, the models most geometrically comparable to other models for the circumstance produced with the Alpha complex have very low values.

The graphs generated using the Vietoris Rips and Čech complexes reveal that the kernel function values are quite similar. The graphs formed by the Witness complex have the most inconsistent similarity values among these four techniques. When examining the underlying geometries of each point cloud, the kernel function assigns large values to very comparable geometries. Even if the Vase 2 model is physically comparable to models 1 and 3, the inputs of the Witness complex’s similarity matrix are relatively high. The observation that the kernel function computed on the graphs produced with the Vietoris Rips and Čech complexes has a large value when it is low is another result of the calculations performed on the graphs acquired with the Alpha complexes.

Considering all of these circumstances, it is feasible to conclude that the 1-skeleton of Alpha complexes are the graphs in which the kernel function developed in this research performs the best. Additionally, it indicates that these values vary when the underlying geometries have sharp edges. Since geodesics become geometrically singular at sharp endpoints, it is said that the presented kernel function provides better geometrically correct results when the Alpha complex is utilized to generate graphs.

**Figure 8:**
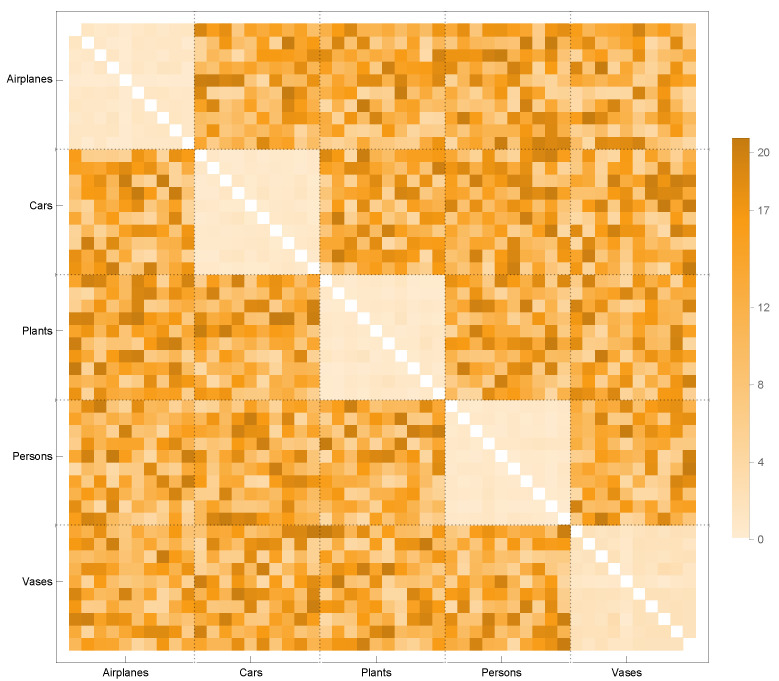
Interclass similarity matrix for graphs obtained by the Alpha complex.

In order to apply this kernel function to assess the similarities between distinct model classes, the similarities between the classes are analyzed in our research using the graphs derived from the Alpha complexes. Within the scope of this precision, Figure 8 depicts the inter-class similarity matrix constructed by applying the kernel function to the graphs generated using the Alpha complexes of five distinct models. This matrix also displays graphs with comparable discrete geodesy distributions whose values are close to zero. There are two remarkable commonalities between the two courses. The Car and Vase models, as well as the Airplane and Person models, are among the most different models. In other words, the kernel function in these models has high values.

### 3.3. Classification

Figure 8 illustrates how useful similarity matrices between classes are when they are produced using the graph kernel function discussed in this study. This part describes how to use this kernel function to infer the class to which a given point cloud belongs. The goal is to create a model that learns from the input graphs $K_{1}^{\left(1\right)},K_{2}^{\left(1\right)},\ldots ,K_{N}^{\left(1\right)}$ and predicts the label of new, unobserved graphs using this dataset of input graphs.

With the usage of graph kernel functions in convolution processes, particularly with the advancements in GCN networks, several deep learning architectures have been built in recent years [74,75,76,77,78]. However, these systems often utilize vectors between graph vertices or edges that correlate with different physical qualities. Additionally, kernel-based classification techniques use kernel functions created across different subgraphs to quantify the similarities between two vertices. It is important to build a matrix between the vertex clusters, namely graph communities, and not between vertex pairs, in the classification process since the kernel function taken into account in this study takes into account the discrete geometries underlying the 3D point clouds.

**Figure 9:**
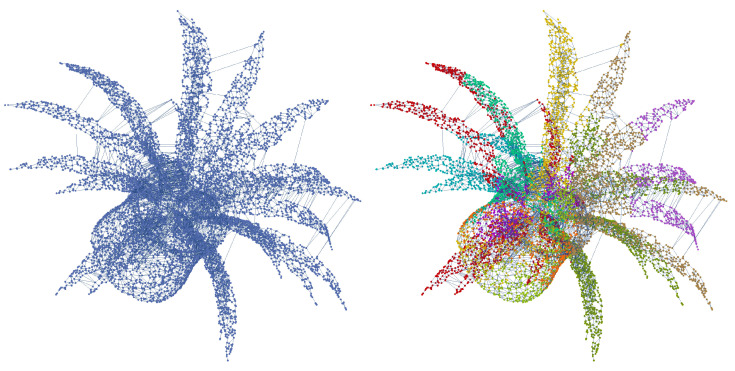
The 1-skeleton of an Alpha complex emerging from plant point cloud and 10 communities obtained using the Fluid Communities approach.

It is known that the matrices of kernel functions created between two graph classes include the structures utilized to embed graphs in Hilbert space. Therefore, it is inappropriate to apply the in-class kernel function directly to solve the graph classification issue. In this study, communities of graphs that are specific to each point cloud were identified, and matrices with the kernel function were produced among the subgraphs generated by the communities. The Fluid Communities [79] approach was used in this study to find the communities of each graph. The propagation mechanism on which Fluid Communities is based is state of the art in terms of computing cost and scalability. Despite being very effective, Fluid Communities can locate communities in artificial graphs with a degree of accuracy that is competitive with the best options. The first propagation-based method that can recognize a changeable number of communities in the network is Fluid Communities. Figure 9 depicts the 10 graph communities found in the 1-skeleton of the Alpha complex using the Fluid Communities approach.

Each initial point cloud sample contains the communities of graphs derived from Alpha complexes for the classification process. The number of communities is determined to be 10, as the community discovery technique utilized in this study needs that number to be predetermined. The kernel function created in the research among the subgraphs discovered by the communities is then used to realize the commonalities between these communities. In order to represent the inherent geometry of each point cloud, a 10×10 matrix is produced. Then, a CNN network with three convolution layers is used. After the first convolution layer, Max Pooling was performed, followed by Average Pooling for the second and third layers. While ReLU activation functions were used for the first two Fully Connected layers, softmax was utilized for classification after the second layer. The ModelNet-40 dataset’s whole sample pool was used in the classification procedure. The ModelNet-40 dataset includes 2468 test models and 9843 training models divided into 40 classes. We uniformly choose 1024 points from each model and normalize them to a unit sphere in order to compare our results with those of other approaches in the literature. We give accurate data from the classification procedure and comparisons to alternative approaches in Table 2.

## 4. Discussion and Conclusions

Graphs are maybe one of the most comprehensive data structures used in machine learning. Complex objects may be represented using graphs as a collection of items and their connections, each of which can be annotated with information such as category or vectorial vertex and edge properties. As graph states, both unstructured vector data and structured data types such as time series, images, volumetric data, point clouds, and asset bags may be understood. Most importantly, the extra flexibility that graph-based representations provide is helpful for a variety of applications.

Kernel approaches are being increasingly used as a method for determining how comparably organized items are. In general, kernel approaches compare two objects using a kernel function that corresponds to an inner product in a kernel Hilbert space. Finding an appropriate kernel function that can be traced computationally while capturing the structure’s semantics is challenging for kernel approaches. In this paper, a kernel function based on the distribution of discrete geodesics on graphs is used to solve the point cloud comparison problem, one of the most important topics in computer vision. Creating this kernel function requires the description of a technique sensitive to isometries and topological transformations.

The dataset was chosen based on the Princeton ModelNet-40 benchmark. Similarity matrices were generated by applying the kernel function to 10 point clouds across five distinct categories. When the similarity matrices are assessed, it is discovered that the graphs of the 1-skeletons of the Alpha complexes provide the highest internal similarity of the categories. Among the point clouds within each category, the similarity value (lower kernel value) between models that are more topologically connected, i.e., structural holes and bottleneck structures, is rather high. The kernel value distributions of the Airplane, Car, Person, Plant, and Vase models attain lower values (more similarity), notably in the Car and Vase models. When the point clouds of both models are analyzed, it becomes evident that these two groups have several topological similarities. Therefore, we may conclude that the kernel function discussed in this research performs well when applied to topological similarities, particularly when the Alpha complex is considered.

The classification of graphs is an issue that regularly arises in machine learning research. Kernel functions that are defined by structural measurements between the related subgraphs of a graph are commonly used for graph classification problems. In this study, the kernel function suggested for graph classification is studied. Within the graphs of each point cloud, the input matrices for a simple convolution neural network model are generated. Utilizing the aggregates of graphs with vertex clusters, the category to which a graph in vector form belongs was determined. For each network model, the Fluid Community approach was utilized to integrate a fixed cluster size while identifying the communities. The kernel function was used to generate inherent similarity matrices between the subgraphs generated by graph communities. This method yielded classification results with an accuracy rating of 91.1%. Even though this percentage is not the greatest reported in the literature, the results were quite effective in comparison to other approaches.

This kernel function, which was designed to compare point clouds with their geometries and topologies, is independent of the dimension of the point cloud, and therefore may be utilized in future research with 2D or higher dimensional point clouds. In addition, it is anticipated that the deep learning architecture used in the classification method provided in this paper will be refined, resulting in improved classification outcomes.

## Figures and Tables

**Table 1:** Comparison for the complex-forming methods.

*K*	Dimension	Time Complexity	Guarantee
Del(P)	$2^{O(|P|)}$	O(|P|+|P|log|P|)	Approx. Geometry
$C_{r}\left(P\right)$	$2^{O(|P|)}$	$O(|P{|}^{d+1})$	Nerve Theorem
$VR_{r}\left(P\right)$	$2^{O(|P|)}$	O(M(|P|))	Approx. $C_{r}\left(P\right)$
W(Q,P)	$2^{O(|Q|)}$	$O\left(\frac{\left|P\right|}{\mu^{d^{2}}}\right)$	Approx. Geometry

**Table 2:** Classification results on ModelNet-40 benchmark.

Method	Input	No. Points	Accuracy
[80]	xyz	1024	% 86.1
[81]	xyz	1024	% 87.1
[82]	xyz	1024	% 87.4
[83]	xyz	1024	% 89.2
[84]	xyz	1024	% 90.0
[85]	xyz	1024	% 90.2
[86]	xyz	1024	% 90.6
[87]	xyz	1024	% 90.7
[88]	xyz	1024	% 91.0
[89]	xyz	1024	% 92.2
[90]	xyz	1024	% 92.3
[91]	xyz	1024	% 93.6
Ours	xyz	1024	% 93.7

## Data Availability

Not applicable.
